# Global health research abstracts: April ‘23

**DOI:** 10.1016/j.afjem.2023.05.004

**Published:** 2023-06-06

**Authors:** Dr. Jonathan Kajjimu

**Affiliations:** Faculty of Medicine, Mbarara University of Science and Technology, Mbarara, Uganda

## Abstract

The African Journal of Emergency Medicine, in partnership with several other regional emergency medicine journals, publishes abstracts from each respective journal. Abstracts are not necessarily linked to open access papers however, all abstracts are accessible without subscription.

## Annals of emergency medicine (North America)

**HIV preexposure prophylaxis in the emergency department: a systematic review.**

Gormley, M. A., Nagy, T. R., Moschella, P., Lu, Z., Rodriguez, J., & Roth, P. (2022). *Annals of Emergency Medicine*.

**DOI:** https://doi.org/10.1016/j.annemergmed.2022.07.015

## Study objective

Many emergency departments (EDs) have identified the importance of HIV prevention and have implemented steps to screen and offer preexposure prophylaxis (PrEP). The objective of this study was to systematically review existing literature that identifies PrEP eligibility in the ED and summarize outcomes along the PrEP cascade of care (awareness, interest, linkage to treatment, initiation, and retention) for patients in ED.

## Methods

Four databases captured all PrEP-related studies in EDs from January 1, 2013 to January 27, 2022. Data were extracted on study characteristics and outcomes, and study quality was assessed using a modified quality assessment tool by the Effective Public Health Practice Project.

## Results

Of the 218 articles, 16 were subjected to full-text review, and 7 met inclusion criteria. Although most studies identified patients who were PrEP eligible using criteria adapted from the 2017 Centers for Disease Control and Prevention PrEP guidelines, the number and time frame for each criterion varied. Six studies reported outcomes on the PrEP cascade of care, showing a relatively high prevalence of awareness and interest but a very low prevalence of linkage and uptake. No studies documented retention in PrEP treatment.

## Conclusion

Although up to a third of patients in ED assessed in the current study were PrEP eligible, less than half of PrEP-eligible participants had prior knowledge of PrEP, and very few who expressed interest in the ED were ultimately linked to PrEP treatment or initiated PrEP. Future research is necessary to identify strategies to increase PrEP education, interest, and linkage to care from the ED.

Reproduced with permission.  

**Canadian Journal of Emergency Medicine (North America)**

**Pediatric emergency department physicians’ perceptions of virtual mental health assessments for urgent needs.**

Stuart, J., Sheridan, N., Cloutier, P., Reid, S., Tse, S., Spettigue, W., ... & Gray, C. (2023). *Canadian Journal of Emergency Medicine*, 1-7.

DOI: https://doi.org/10.1007/s43678-023-00446-w

## Purpose

Pressures related to the COVID-19 pandemic have created the need to develop innovative ways to deliver mental health care, especially for urgent needs. After the launch of a pediatric Emergency Department (ED) Virtual Care service, we aimed to evaluate pediatric ED physicians’ experiences with the use of ED virtual care for mental health assessments.

## Methods

This mixed-methods study was conducted at a pediatric academic health center in Ontario, Canada. Pediatric ED physicians who conducted ED virtual mental health assessments from May to December 2020 were eligible. Participants completed a 22-question novel survey and were invited to participate in a focus group. Descriptive and thematic analyses were used to analyze the data.

## Results

Twenty-nine physicians provided mental health services through the ED virtual care platform. Twenty-five physicians (86% response rate) completed the survey and 3 (10%) participated in a focus group. While many agreed that virtual care benefits patients (67%), key barriers identified included time constraints, lack of mental health clinician support, and uncertainty around the pediatric ED physicians’ role in these types of assessments. Despite these barriers, physicians recognized the potential benefit of the ED virtual care service for mental health assessments and were largely amenable to improving this process should mental health support be available.

## Conclusions

While many physicians agreed that there is a potential benefit of the ED virtual care platform for urgent mental health assessments, time constraints and lack of confidence in providing satisfactory virtual mental health care with minimal mental health support limited its acceptability. These findings can inform the future implementation of mental health services using an innovative virtual ED platform.

Reproduced with permission.  

**Emergencias (Europe)**

**Effects of midazolam vs morphine in patients with acute pulmonary edema with left ventricular systolic dysfunction: a secondary analysis of data from the MIMO trial.**

Domínguez-Rodríguez, A., Hernández-Vaquero, D., Suero-Méndez, C., Burillo-Putze, G., Gil, V., Calvo-Rodríguez, R., ... & Miró, Ò. (2023). *Emergencias: Revista de la Sociedad Espanola de Medicina de Emergencias*, *35*(1), 25-30. https://bit.ly/3FiHf5z

## Objectives

The midazolam vs morphine (MIMO) trial showed that patients treated with midazolam had fewer serious adverse events than those treated with morphine. In many patients with acute pulmonary edema, the left ventricular ejection fraction (LVEF) is preserved, at 50% or higher. We aimed to determine whether left ventricular (LV) systolic dysfunction (D), defined by an LVEF of less than 50%, modifies the protective effect of midazolam vs morphine.

## Material and methods

The MIMO trial randomized 111 patients with acute pulmonary edema to receive intravenous midazolam in 1-mg doses to a maximum of 3 mg (n = 55) or morphine in 2- to 4-mg doses to a maximum of 8 mg (n= 56). We calculated the relative risk (RR) for a serious adverse event in patients with and without systolic LVD.

## Results

LVEF was preserved in 84 (75.7%) of the patients with acute pulmonary edema. In patients with systolic LVD, 4 patients (26.9%) in the midazolam arm vs 6 (50%) in the morphine arm developed serious adverse events (RR, 0.53; 95% CI, 0.2-1.4). In patients without systolic LVD, 6 patients (15%) in the midazolam arm vs 18 (40.9%) in the morphine arm experienced such events (RR, 0.37; 95% CI, 0.16-0.83). The presence of systolic LVD did not modify the protective effect of midazolam on serious adverse effects (P=.57).

## Conclusion

The effect of midazolam vs morphine in protecting against the development of serious adverse events or death is similar in patients with and without systolic LVD.

Reproduced with permission.  

**Emergency Medicine Journal (Europe)**

**Effect of inclined positioning on first-pass success during endotracheal intubation: a systematic review and meta-analysis.**

Turner, J. S., Hunter, B. R., Haseltine, I. D., Motzkus, C. A., DeLuna, H. M., Cooper, D. D., ... & Kirschner, J. M. (2023). *Emergency Medicine Journal*, *40*(4), 293-299.

DOI: http://dx.doi.org/10.1136/emermed-2021-211968

## Background

Endotracheal intubation is a high-risk procedure. Optimisation of all aspects of the procedure, including patient positioning, is important to facilitate success and minimise complications. The objective of this systematic review was to determine the association between inclined patient positioning and first-pass success and other clinically important outcomes among patients undergoing endotracheal intubation.

## Methods

A search of PubMed, CINAHL, SCOPUS, EMBASE and Cochrane, from inception through October 2020 was conducted. Studies were assessed independently by two authors to determine eligibility for inclusion. Included studies were any randomised or observational study that compared supine to inclined patient positioning for endotracheal intubation and assessed one of our predefined outcomes. Simulation studies were excluded. Study results were meta-analysed using a random effects model. The quality of the evidence for outcomes of interest was assessed using the Grading of Recommendations, Assessment, Development and Evaluations approach.

## Results

A total of 5113 studies were identified, of which 10 studies representing 18 371 intubations were included for meta-analysis. There was no statistically significant difference in the primary outcome of first-pass success rate (relative risk 1.02, 95% CI 0.98 to 1.05) or secondary outcomes of oesophageal intubation, glottic view, hypotension, hypoxaemia, mortality or peri-intubation arrest. Likewise, there were no statistically significant differences in any of the outcomes in predefined subgroup analyses of randomised controlled trials, intubations in acute settings or intubations performed with >45 degrees of incline. Overall quality of evidence was rated as low or very low for most outcomes.

## Conclusion

This systematic review and meta-analysis found no evidence of benefit or harm with inclined versus supine patient positioning during endotracheal intubation in any setting.

Reproduced with permission.  

**Hong Kong Journal of Emergency Medicine (Asia)**

**Risk factors for venous thromboembolism after carbon monoxide poisoning: A nationwide population-based study.**

Cho, Y., Lim, T. H., Ko, B. S., Kang, H., Oh, J., & Lee, H. (2023). *Hong Kong Journal of Emergency Medicine*, *30*(2), 79-86.

DOI: https://doi.org/10.1177/1024907921994426

## Introduction

The risk of venous thromboembolism increases after acute carbon monoxide poisoning. However, studies on the characteristics of patients who develop venous thromboembolism after carbon monoxide poisoning are rare. The aim of this study was to identify the risk factors for venous thromboembolism within 3 months after carbon monoxide poisoning.

## Methods

This is a population-based study that employed nationwide claims data from South Korea. Among the carbon monoxide poisoning patients (⩾18 years), the characteristics of the groups with and without venous thromboembolism (pulmonary embolism or deep vein thrombosis) were identified. All the significant variables in the univariable analysis were included in the multivariable logistic regression to determine the risk factors for venous thromboembolism occurrence.

## Results

Among the 24,232 carbon monoxide poisoning patients, 130 subjects developed venous thromboembolism within 90 days of their carbon monoxide poisoning diagnosis. The significant risk factors for venous thromboembolism in the multivariable analysis were age (adjusted odds ratio (aOR) = 1.01; 95% confidence interval (CI) = 1.003–1.03), intensive care unit admission (aOR = 3.80; 95% CI = 2.34–6.12), length of stay (aOR = 1.02; 95% CI = 1.0001–1.04), congestive heart failure (aOR = 2.17; 95% CI = 1.36–3.42), and cancer (aOR = 1.94; 95% CI = 1.10–3.22). The adjusted odds ratios for intensive care unit admission for patients with pulmonary embolism and deep vein thrombosis were 3.05 (95% CI = 1.61–5.61) and 5.60 (95% CI = 2.89–10.90), respectively.

## Conclusion

Patients with older age, intensive care unit admission, a longer length of stay, congestive heart failure, or cancer are at greater risk of developing venous thromboembolism after carbon monoxide poisoning. In particular, intensive care unit admission was the strongest risk factor for venous thromboembolism, pulmonary embolism, and deep vein thrombosis. Monitoring and administering prophylactic treatments to prevent venous thromboembolism would be helpful in high-risk in carbon monoxide poisoning patients.  

Reproduced with permission.

